# Anthrax in the Shaving-Brush—Japanese Shave without Soap

**Published:** 1916-09-09

**Authors:** G. Arbour Stephens

**Affiliations:** 61 Walter Road, Swansea


*To the Editor of* The Hospital.

### Anthrax in the Shaving-brush—Japanese Shave Without Soap.

*To the Editor of* The Hospital.

Sir,—Your comment in this week's issue of The Hospital tends to emphasise the point I raised last June in *Nature*, when I pointed out the benefits of shaving without soap, whereby no brush is required.

It seems somewhat ironical that the Japanese, who shave without soap or brushes, should have sent over to this country such cheap and nasty articles for which they have no use. G. Arbour Stephens.

61 Walter Road, Swansea, September 2, 1916.

[Dr. Stephens omits to point out that water shaving, though it is effective on many skins, entails the use by prudent people of an emollient for the skin after the operation. His second point demonstrates what excellent men of business the Japanese are capable of being. —Ed. The Hospital.]

